# Gene Mutations as Emerging Biomarkers and Therapeutic Targets for Relapsed Acute Myeloid Leukemia

**DOI:** 10.3389/fphar.2017.00897

**Published:** 2017-12-07

**Authors:** Habsah Aziz, Chow Y. Ping, Hamidah Alias, Nurul-Syakima Ab Mutalib, Rahman Jamal

**Affiliations:** ^1^UKM Medical Molecular Biology Institute, Universiti Kebangsaan Malaysia, Kuala Lumpur, Malaysia; ^2^Department of Paediatrics, Faculty of Medicine, Universiti Kebangsaan Malaysia, Kuala Lumpur, Malaysia

**Keywords:** acute myeloid leukemia, mutation, adult, childhood, relapse, biomarker

## Abstract

It is believed that there are key differences in the genomic profile between adult and childhood acute myeloid leukemia (AML). Relapse is the significant contributor of mortality in patients with AML and remains as the leading cause of cancer death among children, posing great challenges in the treatment of AML. The knowledge about the genomic lesions in childhood AML is still premature as most genomic events defined in children were derived from adult cohorts. However, the emerging technologies of next generation sequencing have narrowed the gap of knowledge in the biology of AML by the detection of gene mutations for each sub-type which have led to the improvement in terms of prognostication as well as the use of targeted therapies. In this review, we describe the recent understanding of the genomic landscape including the prevalence of mutation, prognostic impact, and targeted therapies that will provide an insight into the pathogenesis of AML relapse in both adult and childhood cases.

## Introduction

Acute myeloid leukemia (AML) is a blood cancer which characterized by the infiltration of proliferative, clonal, abnormally differentiated, and occasionally poorly differentiated cells of the hematopoietic system (Döhner et al., 2015), as a consequence of arrested myeloid differentiation. The biology of AML is associated with its age variation, as evidenced by the significant variability of genomic alterations in AML from infancy to adulthood. There is also a significant age-based incidence, with elevated incidence reported in both infants and older adults (Meshinchi and Arceci, 2007; Pui et al., 2011; Jay and Schiffer, 2012; Tasian et al., 2014; Tarlock and Meshinchi, 2015).

AML is frequently diagnosed in very young children and comprises of nearly 25% of childhood leukemias. Nearly 800 cases of children and adolescents are diagnosed with AML in the United States annually (Meshinchi and Arceci, 2007; Pui et al., 2011; Tasian et al., 2014). Adults have a higher incidence and AML is generally considered as a disease of the elderly with a median age at diagnosis of around 70 years (Jay and Schiffer, 2012; Tarlock and Meshinchi, 2015). The incidence increases with age, evidenced by 1.3 per 100 000 population in patients aged less than 65 years old as compared to 12.2 per 100 000 population in patients aged over 65 years old (De Kouchkovsky and Abdul-Hay, 2016). Approximately 19,950 new AML cases were diagnosed and 10,430 patients succumbed to the disease in the USA in 2016 (Howlader et al., 2017). In Malaysia, 2,477 cases with 1,330 new cases of AML were reported for a period of 5 years beginning 2007 until 2011 (Ab Manan et al., 2016). As such, new treatment strategies are urgently needed to improve the patients' survival outcome.

The treatment of AML has not changed drastically since 30 years ago (Döhner et al., 2010), and only 60–70% of childhood AML patients achieved long-term cure with the current intensive cytotoxic chemotherapy regimens (Pui et al., 2011; Moore et al., 2013). The percentage of survival is even lower in adult patients aged 60 years or younger, ranging from 35 to 40%. The cure rate is only 5 to 15% for patients aged above 60 years (Döhner et al., 2010, 2015). Morbidity and mortality in patients with AML is significantly contributed by the primary chemo-refractory disease and relapses. Hence better understanding of the genetic lesions underpinning refractory and relapsed AML is pivotal for developing new therapeutic strategies.

## Relapsed acute myeloid leukemia

Relapse after achieving remission remains as one of the major obstacles in improving the patients' overall survival rate and to achieve long term survival for patients with AML. In adult AML, recurrence usually occurs within 3 years post-treatment in most patients (Döhner et al., 2015). Based on the study carried out by the Eastern Cooperative Oncology Group (ECOG) between 1983 and 1997 on newly diagnosed adult AML patients aged >55 years, 65% of them relapsed and had a median survival of 4.7 months, with only 6% who survived more than 5 years. In contrast, better outcome was reported in younger adult patients (aged < 55 years) in which only 35% of them relapsed (Rowe et al., 2005). The poorer outcome in older patients may be partly attributed to their lower tolerance to side effects induced by intensive chemotherapies (Döhner et al., 2015).

Meanwhile, the median time to relapse in childhood AML has been rather stable for consecutive decades (1976–1991:0.93 years, 1991–1997:0.76 years, 2002–2008:0.8 years), (Rubnitz et al., 2014) and relapsed AML remains as the leading cause of cancer deaths among children accounting for more than 50% of childhood leukemia-related deaths (Moore et al., 2013). The response rate to induction chemotherapy in children with AML is relatively higher than adults. This may be due to the children having a higher tolerance level against intensive chemotherapy, lower prevalence of co-morbidities and more intensive supportive care measures (Tasian et al., 2014).

The differences between childhood and adult AML can be explained by the distinct inherent biology of the disease which is inclusive of the discordant incidences of leukemia-associated genetic alterations, pattern of epigenetic changes, and rates of remission induction (Radhi et al., 2010; Creutzig et al., 2012; Puumala et al., 2013; Schuback et al., 2013). The data derived from studies involving both groups of AML patients suggested that there is a pressing need to decrease the number of cases who will relapse and to improve cure rates.

Certainly, major improvement in the treatment strategies is required to improve the success rate for relapsed AML. There is no specific tailored therapy that suitable for every patient. The treatment of relapsed AML is rather varied and dependent on several factors such as age, general health status, remission duration, and genetic aberrations. In the adult setting, most of the relapsed AML patients will be administered with intensified salvage regimens such as MEC (mitoxantrone, etoposide, cytarabine), or FLAG-IDA (fludarabine, granulocyte colony-stimulating factor, idarubicin) followed by allogeneic hematopoietic stem cell transplant (HSCT) whereas low intensity therapy or best supportive care will be offered to adult patients who are physically weak and could not tolerate high dose therapies (Döhner et al., 2015). For children with relapsed AML, reinduction with chemotherapy using FLAG is commonly practiced with good early response by adding the liposomal daunorubicin to FLAG (Kaspers et al., 2013; Creutzig et al., 2014). Remarkably, CBF-AML relapsed patients who received FLAG plus liposomal daunorubicin had a significantly better 4-year overall survival (82 vs. 58%) as compared to those who received FLAG alone (Kaspers et al., 2013). Similar to adult AML, allogeneic HSCT is offered when suitable matched donor is available and the patient is in remission.

Thus far, the prognosis of relapsed AML remains dismal even with allogeneic HSCT (Thol et al., 2015). It is believed that the leukemia stem cells (LSCs) are the source of chemotherapy resistance and likely responsible for the relapse. The LSCs are similar in characteristics to hematopoietic stem cells (HSCs) but they give rise to undifferentiated leukemic blasts (Guzman et al., 2007a). Liran et al. identified pre-leukemic HSCs in the remission sample of an AML patient that harbored *DNMT3A* mutation. Unlike the AML blasts, these pre-leukemic HSCs survived the induction chemotherapy (Shlush et al., 2014). Few agents, including parthenolide (PTL) (Guzman et al., 2005, 2007b), 4-benzyl-2-methyl-1,2,4-thiadiazolidine-3,5-dione (TDZD-8) (Guzman et al., 2007a), and Fenretinide (Zhang et al., 2013) have been shown to be effective in eradicating LSCs by targeting the LSCs enriched CD34^+^CD38^−^ population. Further studies are warranted to evaluate the effectiveness of these agents as the treatment regimens for relapsed AML.

The genome profiling of matched de novo and relapsed AML by whole genome sequencing (WGS) has revealed the existence of two major patterns of clonal evolution underlying AML progression. The first pattern suggested that the founding clone gained additional mutations and evolved into the relapse clone, whereas the second pattern suggested that the subclone of the founding clone which survived initial therapy gained additional mutations and expanded at relapse (Ding et al., 2012). Therefore, understanding of the AML genome and development of targeted therapies which capable to eliminate both founding clones and subclones is key to improving the survival of relapsed AML.

## Commonly mutated genes in AML and relapse prognosis

Cytogenetic profiling remains as the gold standard for guiding risk-adapted treatment plan in AML patients. However, the high relapse risk among AML patients suggested that a more defined risk stratification strategy and better treatment regimens are needed. In recent years, a long list of mutated genes was identified through various sequencing and genotyping approaches. In this review, we describe the prevalence and prognostic impact of genes which are frequently mutated in both childhood and adult AML according to the gene function categories. The prevalence and prognostic impact from various studies are illustrated in Tables 1, 2.

**Table 1:** The Prevalence of gene mutation in AML according to functional categories.

**Gene**	**Technique (n)**	**Sample size (*n*)**	**Frequency of mutation (%)**		**Region**	**References**
			**Adult**	**Childhood**		
**NUCLEOPLASMIN**						
*NPMI*	PCR & Direct Sequencing	300	48.0		Germany	Döhner et al., 2005
	PCR & Direct Sequencing	295		7.8	USA	Brown et al., 2007
	WGS (1) Genotyping (187)	188	23.9		USA	Mardis et al., 2009
	Targeted Sequencing	195		11.3	Germany	Damm et al., 2011
	Direct Sequencing (190) & Amplicon Deep Sequencing (810)	1000	29.2		Germany	Grossmann et al., 2012
	WGS (50) & WES (150)	200	27.0		USA	Cancer Genome Atlas Research, 2013
	PCR & Direct Sequencing	206		4.0	China	Liang et al., 2013
	PCR & Direct Sequencing	216		4.2	Argentina	Rubio et al., 2016
	WES (22) & Targeted Deep Sequencing (182)	204		3.4	Japan	Shiba et al., 2016
**DNA METHYLATION**						
*DNMT3A*	WGS (1) Targeted Sequencing (280)	281	22.1		USA	Ley et al., 2010
	PCR & Direct Sequencing	180		0	USA	Ho et al., 2011
	PCR & Direct Sequencing	195		1.0	Germany	Thol et al., 2011
	PCR & Direct Sequencing	206		1.2	China	Liang et al., 2013
	WGS (50) & WES (150)	200	26.0		USA	Cancer Genome Atlas Research, 2013
	Direct Sequencing	71	24.0		Canada	Shlush et al., 2014
*IDH1*	PCR & Direct Sequencing	61	7.6		Germany	Paschka et al., 2010
	WGS (1) Genotyping (187)	188	8.5		USA	Mardis et al., 2009
	PCR & Direct Sequencing	257	4.4		USA	Ho et al., 2010
	PCR & Direct Sequencing	274		0	USA	Ho et al., 2010
	PCR & Direct Sequencing	227		1.3	USA	Andersson et al., 2011
	PCR & Direct Sequencing	206		1.1	China	Liang et al., 2013
*IDH2*	PCR & Direct Sequencing	70	8.7		Germany	Paschka et al., 2010
	PCR & Direct Sequencing	227		2.2	USA	Andersson et al., 2011
	PCR & Direct Sequencing	180		2.2	USA	Ho et al., 2011
	PCR & Direct Sequencing	206		0.6	China	Liang et al., 2013
*IDH1 & IDH2*	PCR & Direct Sequencing	459		4.0	Germany	Damm et al., 2011
	WGS (50) & WES (150)	200	20.0		USA	Cancer Genome Atlas Research, 2013
*TET2*	PCR & Direct Sequencing	169		6.5	USA	Kutny et al., 2015
	PCR & Direct Sequencing	104		3.8	Netherlands	Langemeijer et al., 2011
	Amplicon Deep Sequencing	318	27.4		Germany	Weissmann et al., 2012
	PCR & Direct Sequencing	206		1.7	China	Liang et al., 2013
	WGS (50) & WES (150)	200	8.0		USA	Cancer Genome Atlas Research, 2013
**ACTIVATED SIGNALING PATHWAY**						
*FLT3-ITD*	PCR & Direct Sequencing	160	32	21	Netherlands	Cloos et al., 2006—Diagnosis Sample
			37	19	Netherlands	Cloos et al., 2006—Relapse Sample
*FLT3-ITD*	Targeted sequencing	195		12.8	Germany	Thol et al., 2011
*FLT3-ITD*	Direct Ssequencing (190) & Amplicon Deep Sequencing (810)	1000	15.9		Germany	Grossmann et al., 2012
*FLT3-ITD*	PCR & Direct Sequencing	206	15		China	Liang et al., 2013
*FLT3-TKD*	PCR & Direct Sequencing	206		7.4	China	Liang et al., 2013
*FLT3*	WGS (50) & WES (150)	200	28.0		USA	Cancer Genome Atlas Research, 2013
*FLT3-ITD/FLT3-TKD*	PCR & Direct Sequencing	216		17.9	Argentina	Rubio et al., 2016
*FLT3-ITD*	WES (22) and Targeted Deep Sequencing (182)	204		10.8	Japan	Shiba et al., 2016
*NRAS*	WGS(1) Genotyping (187)	188	9.3		USA	Mardis et al., 2009
*NRAS*	PCR & Direct Sequencing	206		12.0	China	Liang et al., 2013
*K-RAS*	PCR & Direct Sequencing	206		6.9	China	Liang et al., 2013
*NRAS OR KRAS*	WGS (50) & WES (150)	200	12.0		USA	Cancer Genome Atlas Research, 2013
*NRAS*	WES (22) and Targeted Deep Sequencing (182)	204		12.7	Japan	Shiba et al., 2016
*K-RAS*	WES (22) and Targeted Deep Sequencing (182)	204		3.4	Japan	Shiba et al., 2016
*KIT*	WGS (50) & WES (150)	200	4.0		USA	Cancer Genome Atlas Research, 2013
	PCR & Direct Sequencing	206		12.0	China	Liang et al., 2013
	WES (22) and Targeted Deep Sequencing (182)	204		20.1	Japan	Shiba et al., 2016
**MYELOID TRANSCRIPTION FACTOR**						
*CEBPA*	Direct Sequencing (190) & Amplicon Deep Sequencing (810)	1,000	7.5		Germany	Grossmann et al., 2012
	WGS (50) & WES (150)	200	6.0		USA	Cancer Genome Atlas Research, 2013
	PCR & Direct Sequencing	206		7.0	China	Liang et al., 2013
	PCR & Direct Sequencing	216		1.9	Argentina	Rubio et al., 2016
	WES (22) and Targeted Deep Sequencing (182)	204		11	Japan	Shiba et al., 2016
*RUNX1*	PCR & Direct Sequencing	945	5.6		Germany	Gaidzik et al., 2011
	Direct Sequencing (190) & Amplicon Deep Sequencing (810)	1,000	17.9		Germany	Grossmann et al., 2012
	WGS (50) & WES (150)	200	10.0		USA	Cancer Genome Atlas Research, 2013
	PCR & Direct Sequencing	206		1.3	China	Liang et al., 2013
	PCR & Direct Sequencing	178		5.6	Iraq & jordan	Al-Kzayer et al., 2014
**CHROMATIN REMODELING**						
*ASXL1*	Direct Sequencing (190) & Amplicon Deep Sequencing (810)	1,000	15.4		Germany	Grossmann et al., 2012
	PCR & Direct Sequencing	740	17.2		Germany	Schnittger et al., 2012
	PCR & Direct Sequencing	206		1.1	China	Liang et al., 2013
*ASXL2*	WES (3) & Target Sequencing (110)	35		25.7	France	Micol et al., 2014
*ASXL2*	WES (3) & Target Sequencing (110)	75	21.3		France	Micol et al., 2014
*ASXL1/ASXL2*	WES (22) and Targeted Deep Sequencing (182)	204		8.8	Japan	Shiba et al., 2016
*BCOR*	WES (1), Amplicon Deep-Sequencing (200) & Direct Sequencing (353)	553	3.8		Germany (200) & italy (353)	Grossmann et al., 2011
	WES (22) & Targeted Deep Sequencing (182)	204		3.4	Japan	Shiba et al., 2016
*MLL-PTD*	Direct Sequencing (190) & Amplicon Deep Sequencing (810)	1000	6.0		Germany	Grossmann et al., 2012
	PCR And Direct Sequencing	206		1.9	China	Liang et al., 2013
*EZH2*	WES (22) and Targeted Deep Sequencing (182)	204		0.98	Japan	Shiba et al., 2016
	Direct Sequencing	128	2.0		USA	Khan et al., 2013
**COHESIN COMPLEX**						
*RAD21, SMC3 & STAG2*	WES (22) and Targeted Deep Sequencing (182)	204		8.3	Japan	Shiba et al., 2016
*SMC3*	WGS	16			USA	Ding et al., 2012
**TUMOUR SUPPRESSOR**						
*TP53*	PCR & Direct Sequencing	235	14		Germany	Haferlach et al., 2008
	Direct Sequencing (190) & Amplicon Deep Sequencing (810)	1,000	11.5		Germany	Grossmann et al., 2012
	WGS (50) & WES (150)	200	8.0		USA	Cancer Genome Atlas Research, 2013
	PCR & Direct Sequencing	206		1.1	China	Liang et al., 2013
	PCR & Direct Sequencing	67	7.8		China	Kao et al., 2014
*WT1*	WGS	24	12.5		USA	Welch et al., 2012
	WGS (50) & WES (150)	200	6.0		USA	Cancer Genome Atlas Research, 2013
	PCR & Direct Sequencing	206		5.8	China	Liang et al., 2013
	WES (22) and Targeted Deep Sequencing (182)	204		7.8	Japan	Shiba et al., 2016
*PHF6*	PCR & Direct Sequencing	353	3		USA	Van Vlierberghe et al., 2011
	WGS	24	8.3		USA	Welch et al., 2012
	WGS	16			USA	Ding et al., 2012

**Table 2:** Prognostic impact of mutated gene in AML.

**Gene**	**Prognostic impact**	**References**
*NPMI*	Favorable prognosis with a reduced risk of relapse	Döhner et al., 2005; Papaemmanuil et al., 2016
*DNMT3A*	Unfavorable outcomes and higher relapse rates Worse prognosis was observed in CN-AML patients	Marková et al., 2012; Patel et al., 2012; Shivarov et al., 2013; Tie et al., 2014
*IDH1* and *IDH2*	Unfavorable outcomes in patients with *IDH1* mutations compared to with *IDH2* mutations Shorter relapse free survival in patients with double positive *IDH* and *IDH* and *NPM1* mutations, but *FLT3*-ITD-negative	Abbas et al., 2010; Paschka et al., 2010; Patel et al., 2012; Aref et al., 2015
*TET2*	The prognostic effect remains controversial.	Gaidzik et al., 2012; Patel et al., 2012; Ahn et al., 2015; Kutny et al., 2015
*FLT3-ITD*	Poor outcomes and high relapse rate	Kottaridis et al., 2002; Shih et al., 2002; Cloos et al., 2006; Alvarado et al., 2014
*FLT3-TKD*	Prognosis value remains unclear and contradicting	Martelli et al., 2013; Ofran and Rowe, 2013
*NRAS*	No difference outcomes in patients with mutant and wild-type NRAS	Bowen et al., 2005; Bacher et al., 2006; Berman et al., 2011
*KIT*	Conferred increased relapse risk in adult CBF-AML with t(8;21) but no significant impact on childhood CBF-AML patients	Pollard et al., 2010; Chen et al., 2016
*CEBPA*	Good prognosis marker with significant longer relapse free overall survival especially in CN-AML Prolonged survival after relapse if patients acquired *CEBPA* mutation during relapse	Renneville et al., 2009a; Pastore et al., 2014; Li et al., 2015; Tawana et al., 2015
*RUNX1*	Unfavorable outcome, predictive of chemotherapy resistance and increased relapse rate	Gaidzik et al., 2011; Grossmann et al., 2012; Ismael et al., 2014
*ASXL1* and *ASXL2*	Predictive of inferior prognosis specifically to male adults with MDS, age and positive *RUNX1* mutations Prognostic implication remains inconclusive in childhood AML	Schnittger et al., 2012; Micol et al., 2014; Döhner et al., 2015; Shiba et al., 2016
*BCOR*	Associated with poorer prognosis in adult AML Similar 3-year overall survival in childhood AML with or without *BCOR* mutation	Grossmann et al., 2011; Shiba et al., 2016
*KMT2A/MLL-PTD*	Conferred an inferior prognosis, especially those with CN-AML Worst prognosis in patients with double positive *IDH* and *DNMT3A* mutations	Döhner et al., 2002; Grossmann et al., 2012; Kao et al., 2015
*EZH2*	Poor prognosis and inferior survival	Kawahara et al., 2012; Larsson et al., 2014
*SMC3, RAD21* and *STAG2*	No association between the mutations and overall survival rate	Shiba et al., 2016
*TP53*	Associated with inferior prognosis and higher relapse risk, the worst prognosis in AML	Grossmann et al., 2012; Hou et al., 2015
*WT1*	Adverse outcomes in both childhood and adult AML, low overall survival rate and high relapse rate	Hollink et al., 2009; Renneville et al., 2009b; Hou et al., 2010
*PHF6*	Poor outcomes in intermediate risk group AML patients Predictive biomarker for relapse SMC3relapse	Ding et al., 2012; Patel et al., 2012

### Nucleophosmin

The nucleophosmin (NPM1) gene encodes for a phosphoprotein (Döhner et al., 2015; Tarlock and Meshinchi, 2015) which is involved in the biogenesis of ribosome, duplication of centrosome during mitosis, cell proliferation, and apoptosis induction through p53 and p19Arf (Falini et al., 2007). *NPM1* mutants have been shown to cause aberrant cytoplasmic localization of *NPM1* and NPM1-interacting proteins, as well as impaired function of the nucleolar wild-type NPM1 protein (Döhner et al., 2015; Tarlock and Meshinchi, 2015). *NPM1* was found commonly mutated in both adult and childhood AML patients, with a higher incidence reported in adults (24–29%) (Mardis et al., 2009; Grossmann et al., 2012; Cancer Genome Atlas Research, 2013) than in children (3–11%) (Brown et al., 2007; Thol et al., 2011; Liang et al., 2013; Rubio et al., 2016; Shiba et al., 2016). The incidence appeared to be higher in cytogenetically normal AML (CN-AML), with 48% in adults (Döhner et al., 2005) and 15% in childhood AML (Rubio et al., 2016). *NPM1* mutations have also been shown to be predictive of a favorable prognosis with a reduced risk of relapse in AML patients (Döhner et al., 2005; Papaemmanuil et al., 2016).

### DNA methylation

DNA methyltransferase 3 alpha (*DNMT3A*), isocitrate dehydrogenase 1 and 2 (*IDH1* and *IDH2*) and tet methylcytosine dioxygenase 2 (*TET2*) are involved in regulating the methylation of the genome (Langemeijer et al., 2011; Shah and Licht, 2011; Lu et al., 2012; Liang et al., 2013; Aslanyan et al., 2014; Ibrahem et al., 2014). DNA methyltransferases (DNMTs) act as a catalyst to convert cytosine to 5-methylcytosine (Shah and Licht, 2011). *DNMT3A* mutation was first identified in an AML patient by WGS (Ley et al., 2010). This mutation is rare in childhood AML, ranging from 0 to 2% (Ho et al., 2011; Thol et al., 2011; Liang et al., 2013) but the occurrence rate has been reported in 22–26% of adult cases (Ley et al., 2010; Cancer Genome Atlas Research, 2013; Shlush et al., 2014). Patients who harbored *DNMT3A* mutations showed unfavorable outcomes and higher relapse rates (Marková et al., 2012). An even worse prognosis was observed in CN-AML patients with this mutation and high dose of anthracycline chemotherapy has been recommended for this subgroup of patients (Marková et al., 2012; Patel et al., 2012; Shivarov et al., 2013). A meta-analysis involving 12 studies with a total of 6,377 patients with *DNMT3A* mutations showed poor prognostic impact on the overall survival, relapse free survival, and event free survival (Tie et al., 2014).

*IDH1* mutations that affect the arginine residue at position R132 or R170, and *IDH2* at R140 or R172 (Abbas et al., 2010; Radhi et al., 2010; Green et al., 2011; Rakheja et al., 2012; Shih et al., 2012) will impair histone demethylation (Lu et al., 2012). *IDH1* mutation was first discovered in 2009 by the WGS in an adult AML genome (Mardis et al., 2009). *IDH1* and *IDH2* mutations were rarely detected in childhood AML, with a frequency of 0–1 and 1–2% respectively, but a higher incidence was seen in children with CN-AML (Ho et al., 2010, 2011; Andersson et al., 2011; Damm et al., 2011; Liang et al., 2013). In contrast, *IDH1* and *IDH2* were detected more commonly in adult AML, with a prevalence of 4–9 and 8–19% respectively (Mardis et al., 2009; Ho et al., 2010; Döhner et al., 2015). Remarkably, a higher frequency of *IDH1/IDH2* (20%) was detected in adult AML via the WGS and WES approach (Cancer Genome Atlas Research, 2013), suggesting NGS could be a more sensitive approach in detecting subclonal mutations in heterogenous AML blast cells. Notably, patients with *IDH1* mutations appeared to have more unfavorable outcomes compared to those with *IDH2* mutations (Abbas et al., 2010; Patel et al., 2012) and shorter overall survival compared to patients without IDH mutation (Aref et al., 2015). Moreover, patients with double positive *IDH* and *NPM1* mutations, but *FLT3*-ITD-negative, showed a shorter relapse free survival (Paschka et al., 2010).

*TET2* converts methylcytosine to 5-hydroxymethylcytosine and has a role in regulating myelopoiesis (Shih et al., 2012). Loss of function mutation in *TET2* decreased DNA hydroxymethylation (Aslanyan et al., 2014). *TET2* was shown to be frequently mutated in adult AML (8–28%) as compared to childhood AML (1–7%) (Langemeijer et al., 2011; Weissmann et al., 2012; Cancer Genome Atlas Research, 2013; Liang et al., 2013; Kao et al., 2014; Kutny et al., 2015). The prognostic effect of *TET2* mutation remains controversial. No significant impact on relapse free survival was observed in a large cohort of younger adult AML patients (Gaidzik et al., 2012) and no difference in relapse incidence at 5 years in CN-AML patients with or without *TET2* mutations (Ahn et al., 2015), whilst other studies demonstrated that *TET2* mutations were correlated with inferior outcomes in favorable-risk CN-AML (Patel et al., 2012) and failure to achieve complete remission in childhood AML (Kutny et al., 2015).

### Activated signaling pathway

Fms-like Tyrosine Kinase 3 (*FLT3*) encodes for a receptor tyrosine kinase which is a membrane-bound receptor with an intrinsic tyrosine kinase domain. Internal tandem duplication (ITD) of the juxtamembrane domain and point mutations of the tyrosine kinase domain (TKD) in *FLT3* have been shown to result in the constitutive activation of the receptor kinase activity (Nakao et al., 1996; Tarlock and Meshinchi, 2015). In both adult and childhood AML, the incidence of *FLT3 ITD* (15–37 and 10–21% respectively) was higher compared to *FLT3 TKD* (10 and 7%) (Cloos et al., 2006; Thol et al., 2011; Damm et al., 2012; Grossmann et al., 2012; Cancer Genome Atlas Research, 2013; Liang et al., 2013; Kao et al., 2014; Rubio et al., 2016; Shiba et al., 2016). *FLT3-ITD* was associated with poor outcomes and high relapse rate, and the duplication has been shown to persist in both diagnosis and relapse samples with no significant changes in frequency (Kottaridis et al., 2002; Shih et al., 2002; Cloos et al., 2006). Even though allogeneic stem cell transplant or the use of FLT3 inhibitors has been proposed as promising treatment approaches for patients harboring *FLT3* mutations, the results have been disappointing due to the high relapse rate (Alvarado et al., 2014). Recently, some studies showed that patients with *FLT3-ITD* AML should be offered allogeneic stem cell transplant when feasible as the preferred post-remission treatment for better survival outcomes (Ho et al., 2016; Oran et al., 2016). Meanwhile, mutations in *FLT3-TKD* appeared to be less commonly detected and its prognosis value remains unclear and contradicting (Martelli et al., 2013; Ofran and Rowe, 2013).

Neuroblastoma RAS Viral (v-ras) Oncogene Homolog (*NRAS*) and Kirsten Rat Sarcoma Viral Oncogene Homolog (*KRAS*) belong to the RAS GTPase family that encode a membrane-associated guanosine nucleotide phosphate (GTP) binding proteins, and are involved in regulating signal transduction upon binding of ligand to a variety of membrane receptors (Bowen et al., 2005; Berman et al., 2011). The occurrence of *NRAS* mutations has been reported among adult (9–12%) and childhood AML (12–13%) but *KRAS* mutations appeared to be more prevalent in adult AML than childhood AML (12 vs. 3–7%) (Mardis et al., 2009; Cancer Genome Atlas Research, 2013; Liang et al., 2013; Kao et al., 2014; Shiba et al., 2016). Larger cohort studies have shown that the clinical outcomes of patients who harbored mutant and wild-type NRAS did not differ, as evidenced by similar complete remission (Bowen et al., 2005; Bacher et al., 2006; Berman et al., 2011) and relapse rates (Bowen et al., 2005; Bacher et al., 2006; Berman et al., 2011).

Proto-Oncogene Receptor Tyrosine Kinase (*KIT*) is involved in hematopoiesis as well as proliferation and regulation of cell survival. The frequency of *KIT* mutation in adult AML was reported lower as compared to childhood AML (4 vs. 12–20%) (Cancer Genome Atlas Research, 2013; Liang et al., 2013; Shiba et al., 2016). A higher frequency was reported in core-binding factor acute myeloid leukemia (CBF-AML) with inv(16) and t(8;21), in both children (19–44%) and adults (16–46%) (Jones et al., 2010; Pollard et al., 2010). Based on the meta-analysis conducted by Chen et al. *KIT* mutations conferred increased relapse risk in adult CBF-AML with t(8;21) (Chen et al., 2016). In contrast, *KIT* mutations did not show significant impact on relapse risk in childhood CBF-AML patients (Pollard et al., 2010). *In vitro* studies demonstrated that the use of a tyrosine kinase inhibitor was effective against leukemic cells harboring *KIT* mutations and served as promising therapeutic approach for AML patients.

### Myeloid transcription factor

CCAAT Enhancer Binding Protein Alpha (*CEBPA*) is a transcription factor that is involved in regulating the differentiation of neutrophils. *CEBPA* mutations were mostly located at the N-terminal domain (NTD) and bZip domain (Ho et al., 2009). The frequency of *CEBPA* mutations reported in childhood AML is generally double that seen in adult AML (2–12 vs. 6–7.5%) (Grossmann et al., 2012; Cancer Genome Atlas Research, 2013; Liang et al., 2013; Rubio et al., 2016; Shiba et al., 2016). Biallelic *CEBPA* mutations are associated with favorable prognosis in CN-AML whilst monoallelic mutations showed contradicting outcomes in patients (Pastore et al., 2014; Li et al., 2015). Several lines of evidence have shown that the *CEBPA* mutation served as a good prognosis marker of AML in which patients with *CEBPA* mutation had significantly longer relapse free overall survival (Renneville et al., 2009a). More strikingly, patients who acquired *CEBPA* mutation during relapse (absent in diagnostic sample) had favorable outcomes with prolonged survival after relapse, with a 67% 10-year overall survival rate (Tawana et al., 2015).

Runt Related Transcription Factor (*RUNX1*) encodes for a core-binding factor which binds to the core element of many enhancers and promoters. *RUNX1* mutations are more prevalent in AML without complex karyotype, with 5–18% in adult AML (Gaidzik et al., 2011; Grossmann et al., 2012; Cancer Genome Atlas Research, 2013) and 1–6% in childhood AML (Liang et al., 2013; Al-Kzayer et al., 2014). *RUNX1* mutation is associated with unfavorable outcome, is predictive of chemotherapy resistance with a refractory rate of 30% and also an increased relapse rate (Gaidzik et al., 2011; Grossmann et al., 2012). Clonal evolution of *RUNX1* mutation was reported in the relapsed samples of childhood AML cases (Ismael et al., 2014). Clinical analysis has proven that patients with *RUNX1* mutations who received allogeneic HSCT had a 52% 4-year relapse free survival as compared to those treated with conventional intensive post-remission therapy (0% 4-year relapse free survival) (Gaidzik et al., 2011). Hence, allogeneic hematopoietic stem cell transplant (HSCT) is highly recommended for patients harboring RUNX1 mutation as compared to conventional consolidation therapy.

### Chromatin remodeling

Additional Sex Combs Like 1 Transcriptional Regulator (*ASXL1*) and Additional Sex Combs Like 2 Transcriptional Regulator (*ASXL2*) encodes a dual-function chromatin-binding protein, which acts as a transcription activator or repressor (Katoh and Katoh, 2003). *ASXL1* mutation is frequently associated with secondary AML evolving from myelodysplastic syndrome (MDS) (Devillier et al., 2012) with a 5–17% occurrence in adult AML, with a higher frequency in cases with intermediate risk cytogenetics (31%) and CN-AML (13%) (Grossmann et al., 2012; Schnittger et al., 2012; Döhner et al., 2015). The incidence of *ASXL1* mutation is rare (1%) in childhood AML and increases with age (Liang et al., 2013). However, the prevalence of *ASXL2* mutation in both adult (22%) and childhood (26%) AML is almost similar (Micol et al., 2014). In childhood AML, the prognostic implication of *ASXL1* and *ASXL2* mutations remains inconclusive. Shiba et al. (2016) did not observe the association with inferior outcome, whilst Micol et al. (2014) reported that the mutations were associated with increased relapse risk. Thus, a larger study cohort is needed to confirm the prognostic effect of *ASXL1* and *ASXL2* in childhood AML. Meanwhile, *ASXL1* mutations were predictive of inferior prognosis and contributed significantly to leukemogenesis in male adults with AML with MDS, those older in age and those harboring *RUNX1* mutations (Schnittger et al., 2012; Döhner et al., 2015).

BCL6 Corepressor (*BCOR*) encodes a POZ/zinc finger transcriptional repressor (Huynh et al., 2000), and its loss-of-function mutation has led to the inhibition of proliferation and differentiation of myeloid cells (Cao et al., 2016). The occurrence rate of *BCOR* mutation in both childhood and adult AML was found to be almost similar, namely 3.4 and 3.8% (Grossmann et al., 2011; Shiba et al., 2016) respectively by using the WES technique. The frequency of *BCOR* mutations was higher in CN-AML adult patients (17%), and associated with poorer prognosis, with an overall 2-year survival rate of 25.6% (Grossmann et al., 2011). In contrast, the 3-year overall survival was similar for childhood AML patients with or without *BCOR* mutation (71 vs. 72%) (Shiba et al., 2016).

Lysine (K) Methyltransferase 2A (*KMT2A*) or formerly known as Mixed-Lineage Leukemia (*MLL*) is involved in regulating gene expression during early development and hematopoiesis. *MLL-PTD* was found mutated in 6% of adult AML patients (Döhner et al., 2002; Grossmann et al., 2012) and a lower frequency of *MLL-PTD* (2%) was reported in childhood AML (Liang et al., 2013). *MLL-PTD* conferred an inferior prognosis, in which the 3-year event free survival was only 10.5% (Grossmann et al., 2012), and 69% of CN-AML patients with *MLL-PTD* relapsed after achieving remission (Döhner et al., 2002). Therefore, *MLL-PTD* is associated with inferior outcomes in AML patients especially those with CN-AML. In addition, inferior event free survival (median 0 vs. 6.8 months) was noted in *MLL*-PTD AML patients with *DNMT3A* mutations compared to those without *DNMT3A* mutations (Kao et al., 2015).

Enhancer of Zeste Homologue 2 (*EZH2*) is a histone methyltransferase which is involved in transcriptional repression by depositing histone H3 lysine 27 (H3K27) and also plays a pivotal role in hematopoiesis (Yap et al., 2011; Lund et al., 2014). *EZH2* controls the balance between cell differentiation and renewal, thus its dysregulation may lead to tumorigenesis (Lund et al., 2014). *EZH2* mutations decreased H3K27 trimethylation and increased chromatin relaxation. *EZH2* mutations have been detected in ~2% adult AML (Khan et al., 2013) and ~1% childhood AML (Shiba et al., 2016). *EZH2* mutation was more frequently detected in acute megakaryoblastic leukemia (AMKL) and AMKL with Down Syndrome (DS-AMKL), 16 and 33% respectively (Yoshida et al., 2013). *EZH2* mutations was correlated with poor prognosis and inferior survival (Kawahara et al., 2012; Larsson et al., 2014). 3-Deazaneplanocin (DZNep), an EZH2 inhibitor, may serve as a potential drug for consolidation or maintenance therapy in AML patients to decrease the chance of relapse by eliminating the cancer stem cells which are resistant to conventional chemotherapy such as cytarabine and daunorubicin (Miranda et al., 2009; Horton and Huntly, 2012).

### Cohesin complex

Cohesin is a multiprotein complex which comprises of four primary subunits *SMC1A, SMC3, RAD21*, and *STAG1/STAG2*, and is responsible for sister chromatid cohesion, gene expression regulation and DNA repair (Haarhuis et al., 2014; Kim et al., 2016). *SMC3* gene mutation was first described by Ding et al. (2012) by comparing the genomic landscape of a primary and matched relapse adult AML using WGS. The *SMC3* mutation was found enriched in relapsed clones and may be induced by the damaging effects from cytotoxic chemotherapy. Subsequently, in 2016, Shiba et al. reported that *SMC3, RAD21*, and *STAG2* mutations were detected in 8.3% of 204 childhood AML cases, and the truncating mutations resulted in loss of cohesin function. However, they did not observe an association between the mutations and overall survival rate (Shiba et al., 2016). A larger cohort is therefore needed to provide a better overview of the utility of cohesin as a prognostic biomarker.

### Tumor suppressor

Tumor Protein p53 (*TP53*), is the most well-studied tumor suppressor gene and is involved in cell cycle regulation in response to cellular stress. *TP53* is frequently mutated in AML patients with complex karyotypes, with an incidence rate of 69–73% (Haferlach et al., 2008; Grossmann et al., 2012). The incidence rate was reported lower in other cytogenetic subgroups, ranging from 8 to 14% in adult AML and about 1% in childhood AML (Haferlach et al., 2008; Grossmann et al., 2012; Cancer Genome Atlas Research, 2013; Liang et al., 2013; Kao et al., 2014). *TP53* mutation was associated with inferior prognosis and higher relapse risk, in which the 3-year overall survival and event free survival was 0% (Grossmann et al., 2012). The observation of a poorer prognosis for *TP53* mutation was also independent of age, karyotype and other mutations such as *NPM1/FLT3-ITD, CEBPA, RUNX1, WT1, DNMT3A*, and *IDH2* (Hou et al., 2015). Based on above evidence, *TP53* mutation may serve as a promising predictive biomarker of the worst prognosis in AML.

Wilms Tumor 1 (*WT1*) is a transcription factor involved in urogenital development and was found overexpressed in AML with a role in promoting leukemogenesis (Rampal and Figueroa, 2016). The frequency of *WT1* mutation was slightly higher in adult AML compared to childhood AML (6–12.5 vs. 6–8%) (Welch et al., 2012; Cancer Genome Atlas Research, 2013; Liang et al., 2013; Shiba et al., 2016). *WT1* mutation was significantly correlated with adverse outcomes in both childhood and adult AML. In adult AML (*n* = 268, aged 15–50 year), the 4-year overall survival rate was only 22%, and the relapse rate was relatively high (82%) (Renneville et al., 2009b). A similar finding of higher relapse incidence (85.7%) was reported in 470 adult AML patients with *WT1* mutations (Hou et al., 2010). The similar pattern was seen in childhood AML with cumulative relapse incidence of 70% in 298 patients (Hollink et al., 2009).

Plant Homeodomain Finger 6 (*PHF6*) acts as a tumor suppressor gene. *PHF6* non-sense and frameshift mutations have been shown to result in loss-of-function alleles (Van Vlierberghe et al., 2011). *PHF6* mutations were observed in T-cell acute lymphoblastic leukemias, with an incidence rate of 20% (Van Vlierberghe et al., 2010). In contrast, *PHF6* mutations were only detected in 3–8% adult AML (Van Vlierberghe et al., 2011; Ding et al., 2012), and more frequently detected in males than females (Van Vlierberghe et al., 2011). Adverse outcomes had been reported in AML patients with intermediate risk group (Patel et al., 2012). *PHF6* mutations were found to be acquired during initiation of leukemogenesis and were also enriched in relapsed samples (Ding et al., 2012), hence could serve as a potential predictive biomarker of AML relapse.

## Development of targeted therapies in AML

The poor prognosis for AML has been a key driver for the research and development of targeted therapies. The emerging targeted therapies for the management of AML include FLT3 inhibitors, IDH inhibitors, and MEK inhibitors. In April 2017, the new drug midostaurin (Rydapt; Novartis Pharmaceuticals, Inc) received approval by the US Food and Drug Administration (FDA) for the treatment of adult patients with newly diagnosed *FLT3*-mutated AML (Levis, 2017). Midostaurin (N-benzoyl staurosporine also previously known as CGP41251 and PKC412), a derivative of staurosporine, is an indolocarbazole and is a pan-kinase inhibitor, a derivative of staurosporine (Tamaoki et al., 1986). It is a kinase inhibitor against both the *FLT3-ITD* and *FLT3-TKD* mutants, and relapsed/refractory patients treated with midostaurin showed great reduction in peripheral blood and marrow blasts (Stone et al., 2005; Fischer et al., 2010). Midostaurin has been approved and recommended to be given in combination with drugs for induction (cytarabine and daunorubicin) and consolidation (high-dose cytarabine) (Levis, 2017).

Another first generation FLT3 inhibitor, Lestaurtinib (previously known as CEP-701), is a relatively less selective compound compared to midostaurin, and its usage has not been able to improve the survival in *FLT3* mutated AML patients (Knapper et al., 2017). The UK MRC AML15 clinical trial (ISRNCTN17161961) & UK NCRI AML17 (ISRNCTN55675535) involving five hundred patients in the United Kingdom, Denmark, and New Zealand showed no significant differences in the 5-year overall survival and 5-year relapse-free survival when added to the standard chemotherapy for newly diagnosed *FLT3* mutated AML (Knapper et al., 2017). This factor has contributed to the development of second generation of FLT3 inhibitors which are more potent and more selective in action.

Quizartinib (AC220) is a second generation FLT3 inhibitor that exhibits low nanomolar potency in biochemical and cellular assays, and has exceptional kinase selectivity (Zarrinkar et al., 2009). In the phase 3 clinical trial, QuANTUM-First, to test the effect of quizartinib in combination with conventional chemotherapy in *FLT3/ITD* patients (NCT02668653), and the QuANTUM-R trial to compare quizartinib monotherapy vs. conventional salvage therapies among relapsed/refractory *FLT3-ITD* mutated patients (NCT02039726), the results showed a higher composite complete remission (CRc) rate in both older and younger patients with *FLT3-ITD* mutated relapsed/refractory AML (Stein and Tallman, 2016; Fathi and Chen, 2017; Saygin and Carraway, 2017). Another selective FLT3 inhibitor that has been tested in a phase 3 clinical trial is Gliteritinib. Gilteritinib (previously referred to as ASP2215) is a pyrazinecar-boxamide derivative, with activity against both *FLT3-ITD* and *FLT3-TKD* mutations (Lee et al., 2017). Remarkable results were obtained in terms of the CRc among patients with *FLT3-ITD* and *FLT3-TKD* mutations (Perl et al., 2017) and also in the ongoing phase 3 clinical trial to test gilteritinib vs. salvage chemotherapy in relapsed/refractory *FLT3* mutated AML (NCT02421939).

The limiting factor for these second generation FLT3 inhibitors was the short duration of response as shown with quizartinib (3 months) and gilteritinib (5 months) (Saygin and Carraway, 2017). In addition, there have been reports on resistance to quizartinib, which was shown to be attributed by the mutations in the TKD of the FLT3 gene (Moore et al., 2012; Smith et al., 2012). Crenolanib, a highly selective and potent next-generation FLT3 inhibitor, can overcome quizartinib resistance by targetting both *FLT3-ITD* mutants and the *FLT3-D835* point mutants (Galanis et al., 2014). This drug is currently in a phase 3 clinical trial, to test the effect in combination with salvage chemotherapy for relapsed or refractory *FLT3* mutated AML patients (NCT02298166) (Levis, 2017).

Another major breakthrough in the treatment of AML is the approval of enasidenib by the FDA in early August 2017. Enasidenib is approved for the treatment of relapsed or refractory in *IDH2* mutated adult AML patients. Enasidenib (AG-221/CC-90007) is a first-in-class, oral, selective inhibitor of mutant-IDH2 enzymes and assessment outcomes in the largest relapsed or refractory mutant-*IDH2* AML patient shows a 40.3% overall response rate with 9.3 months median overall survival, and 19.7 months overall survival for those who attained complete remission (Stein et al., 2017). Meanwhile, other oral inhibitors such as AG-120, IDH305, and FT-2102 are currently being evaluated in AML patients with *IDH1* mutation. Early results of ongoing clinical trials for the AG-120 ((NCT02074839) and IDH305 (NCT02381886) in relapsed or refractory AML patient showed that both drugs were well-tolerated and the overall response rate was 36 and 33% respectively (Birendra and DiNardo, 2016; Saygin and Carraway, 2017). Therefore, these IDH1 inhibitors seem promising as targeted therapies for AML.

MAP–ERK kinase (MEK) inhibitor has been used in *RAS* mutated AML patients. Selumetinib (AZD6244, ARRY-142886) is a potent and selective small-molecule inhibitor of MEK (Yeh et al., 2007; Adjei et al., 2008). The phase II study of oral selumetinib showed hematologic improvement in platelets count as well as reduction in the bone marrow blasts percentage in AML patients with *KRAS* mutation (Jain et al., 2014). Interestingly, the use of this MEK inhibitor also resulted in good response and disease stabilization in patients with *KIT* mutation (rs3733542 in exon 18) but require further validation (Jain et al., 2014). The favorable effect of selumetinib makes this MEK inhibitor a good potential in targeting the *RAS* mutation in AML patients.

**Figure 1:**
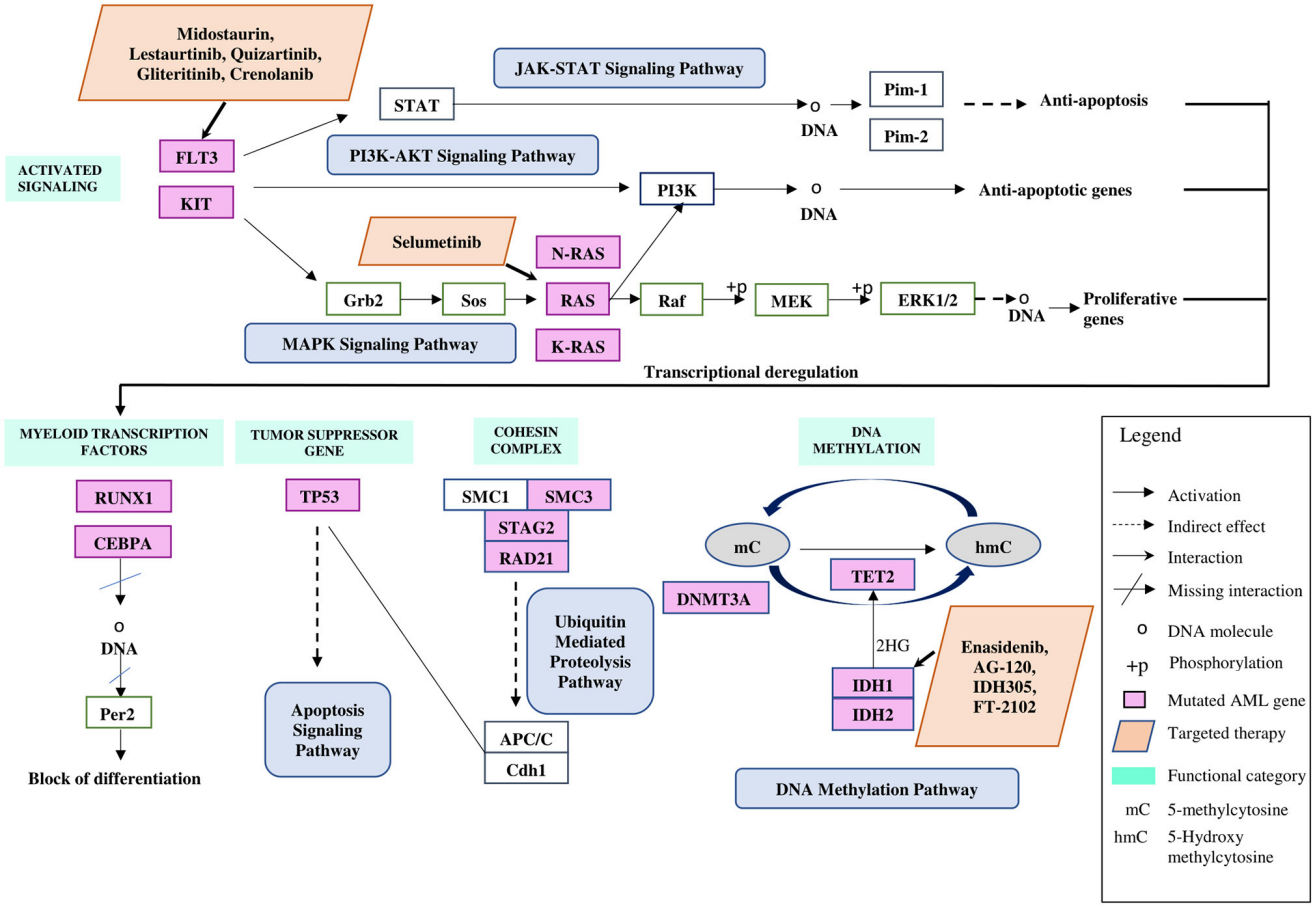
Commonly mutated genes in both childhood and adult AML illustrated based on functional categories, in relation to pathways involved with targeted therapies. Figure was adapted and modified from Kanehisa et al. (2016) and Döhner et al. (2015).

The emergence of these molecular targeted therapies has contributed to the improvement in the treatment of AML patients specifically resulting in better response rate and overall outcomes, with less toxicity than standard cytotoxic therapy. The molecular targeted therapies for the management of AML as discussed in this review are illustrated in Figure 1 and Table 3.

**Table 3:** Targeted therapies in AML.

**Gene**	**Drugs**	**Status**	**Single/Combination**	**References**
*FLT3-ITD/FLT3-TKD*	Midostaurin (CGP41251/PKC412)	FDA Approved (Rydapt; Novartis Pharmaceuticals, Inc)	In combination with induction and consolidation chemotherapy	Levis, 2017
	Lestaurtinib (CEP-701)	Phase 3 Clinical Trial UK MRC AML15 (ISRNCTN17161961) & UK NCRI AML17 (ISRNCTN55675535)	In combination with induction and consolidation chemotherapy	Knapper et al., 2017
	Quizartinib (AC220)	Phase 3 Clinical Trial QuANTUM-First (NCT02668653)	In combination with induction and consolidation chemotherapy	Stein and Tallman, 2016; Fathi and Chen, 2017; Saygin and Carraway, 2017
		Phase 3 Clinical Trial QuANTUM-R (NCT02039726)	Single agent	
	Gliteritinib (ASP2215)	Phase 3 Clinical Trial (NCT02421939)	Single agent	Lee et al., 2017; Perl et al., 2017
	Crenolanib	Phase 3 Clinical Trial (NCT02298166)	In combination with salvage chemotherapy	Galanis et al., 2014; Levis, 2017
*IDH2*	Enasidenib (AG-221/CC-90007)	(Idhifa) FDA Approved (Celgene Corporation)	Single agent	Stein et al., 2017
*IDH1*	AG-120	Phase 1 Clinical Trial (NCT02074839)	Single agent	Birendra and DiNardo, 2016
	IDH305	Phase 1 Clinical Trial (NCT02381886)	In combination with induction and consolidation chemotherapy	Stein and Tallman, 2016; Saygin and Carraway, 2017
	FT-2102	Phase 1/1b Clinical Trial (NCT02719574)	In combination with azacitidine	
*RAS*	Selumetinib (AZD6244, ARRY-142886)	Phase II Consortium (Chicago, IL)	Single agent	Jain et al., 2014

## Conclusion

The breakthrough in genomic technologies, especially NGS, has enhanced our understanding about the genetic landscape of AML, and has led to the discovery of a long list of mutations which are potentially useful as prognostic markers of AML. Interestingly, the prognostic impact of the genetic events from the same functional categories are rather varied. For instance, the presence of *NPMI, IDH2*, and *CEBPA* mutations predicted a favorable prognosis whilst *KRAS* and *NRAS* mutations had little clinical impact. Also, mutations in *DNMT3A, IDH1, TET2, FLT3 ITD, MLL*, and *EZH2* are associated with poorer prognosis, whilst mutations in *RUNX1, WT1*, and *TP53* are predictive of the worst outcomes and increased relapse risk in both adult and childhood AML. Meanwhile, the prognostic impact of *KIT, ASXL1/ASXL2*, and *BCOR* mutations between adult and childhood AML showed a contradiction. The implications of *FLT3-TKD, SMC3, RAD21, STAG2*, and *PHF6* mutations in AML remain unclear and their usefulness as predictive biomarkers of relapse warrants further investigations. Taken together, gene mutations provide additional valuable clinical information which could help to refine the risk or prognostic classification of AML and to guide risk-adapted therapies. Discovery of gene mutations which are uniquely acquired or enriched in relapse AML may pave new ways toward the development of novel therapeutic therapies and precision medicine for AML.

## Author contributions

HAz drafted this manuscript. CP, HAl, N-SA, and RJ involved in the critical evaluation of the manuscript.

### Conflict of interest statement

The authors declare that the research was conducted in the absence of any commercial or financial relationships that could be construed as a potential conflict of interest.
